# Primary intrathoracic liposarcoma: a clinicopathologic study and prognostic analysis of 23 cases

**DOI:** 10.1186/1749-8090-9-119

**Published:** 2014-07-04

**Authors:** Ming Chen, Jun Yang, Lei Zhu, Cao Zhou, Heng Zhao

**Affiliations:** 1 Department of Thoracic Surgery, Shanghai Chest Hospital, School of Medicine, Shanghai Jiao Tong University, 241 West HuanHai Road, Shanghai, China; 2 Department of Pathology Surgery, Shanghai Chest Hospital, School of Medicine, Shanghai Jiao Tong University, Shanghai, China

**Keywords:** Intrathoracic liposarcoma, Histological type, Radical surgery, Overall survival, Disease-free survival

## Abstract

**Background:**

Primary intrathoracic liposarcoma is an extremely rare malignancy as well as a rare histologic subtype of intrathoracic sarcoma. Relatively few reports appear in the world literatures. We explored the clinicopathologic features and prognostic factors of this tumor in this study.

**Methods:**

We retrospectively analyzed the clinicopathological data of 23 patients with primary intrathoracic liposarcoma who were treated in Shanghai chest Hospital affiliated to Jiao Tong University, from January 2003 to March 2013. These patients were classified into three groups according to the distinct tumor locations, including mediastinum, pleura and lung liposarcoma. Also, these patients could be divided into four types, including well-differentiated, myxoid, dedifferentiated and pleomorphic liposarcoma. The influences of age, sex, tumor size, tumor location, tumor histologic type and therapy on the prognosis of the patients were analyzed.

**Results:**

There were no significant difference for survival among distinct liposarcoma locations. However, significant difference for survival among distinct liposarcoma types were observed. Poor disease-free survival (DFS) was observed in the myxoid, pleomorphic and dedifferentiated types as compared to well-differentiated type (*P* = 0.038). Inferior overall-survival (OS) was observed in dedifferentiated, pleomorphic and myxoid types relative to well-differentiated type (*P* = 0.027). The radical surgery was a favorable prognostic factor for OS, as demonstrated by the better OS of the radical surgery group as compared to that of the non-radical surgery group ( *P* = 0.029). Notably, there were no significant differences for DFS and OS in other clinical parameters including tumor size, gender and age. In addition, radiotherapy and/or chemotherapy could not improve the prognosis of the patients receiving non-radical surgery or suffering from relapse.

**Conclusions:**

The histological type and the radical surgery are the factors that influence the behavior and prognosis of liposarcoma. In general, radiotherapy and chemotherapy are believed to be ineffective therapeutic modalities for survival. So it is essential to completely resect the primary intrathoracic liposarcoma as radical cure of the disease.

## Background

Liposarcoma most commonly arises in the retroperitoneum or thigh, but can also involve numerous other anatomic sites, such as the inguinal area, popliteal fossa, neck, as well as the genitourinary and aerodigestive tracts. However, primary intrathoracic liposarcoma is very rare and only a few isolated cases have been reported [1,2].

Liposarcoma has been subclassified histologically into well-differentiated, myxoid, pleomorphic, and dedifferentiated types according to the 2012 NCCN classification of liposarcoma.

According to location classification, mediastinal liposarcomas are extremely rare, making up from 0.1%–0.75% of all mediastinal tumors [3]. The pleural and pulmonary liposarcoma are more scarce location. Hitherto, the prognosis of this group sarcomas for distinct histological subtypes and different locations is still unclear. Therefore, we carried out this study to explore the correlation between the survival and distinct tumor locations/histological types of this rare malignancies for the first time.

## Methods

### Patients

The 23 patients who received primary intrathoracic liposarcoma resection in Shanghai Chest Hospital affiliated to Shanghai Jiao Tong Universityfrom January 2003 to March 2013 were included in this study. All specimens of these patients were confirmed by experienced pathologists.

### Clinical data

In these 23 cases, 10 cases were mediastinal liposarcoma, 9 cases were pleural liposarcoma, and 4 cases were lung liposarcoma. 8 cases were type of well-differentiated liposarcoma, 8 cases were type of myxoid liposarcoma, 4 cases were type of de-differentiated liposarcoma, 3 cases were type of pleomorphic liposarcoma. Of these cases, 12 were males, 11 were females, and the ratio of the male to female was 1.1:1. Patients ranged in age from 16 to 72 years with a median age of 49 years. Tumor size was calculated by the maximum diameter. The median size of these 23 patients was 8 cm (range 4 to 39 cm). Among these cases, tumor sizes of totally11 cases were > 8 cm.

The most common presenting symptom include chest pain, cough, dyspnea on exertion and shortness of breath, on occasion, patients may be asymptomatic. Contrast-enhanced chest CT scans and X-ray were performed for all patients. If the lesion was suspected violations of the heart or great vessels, the MRI examination was performed.

### Therapy

Primary intrathoracic liposarcoma resection was performed in all the 23 patients in this study. All of them underwent median sternotomy or thoracotomy. Lobectomy and systematic lymph node dissection were performed in pulmonary liposarcoma group. Among 17 patients, who received radical surgery, 9 patients suffered from relapse. Among these 9 patients, metastatic cases received chemotherapy or/and radiotherapy. Adjuvant radiotherapy or/and salvage surgery were made depending on individual situation among local recurrence cases (Table 1). Due to advanced disease, 6 cases were received non-radical surgery, along with chemotherapy or/and radiotherapy (Table 2).

**Table 1:** Basic characteristics of 17 patients received complete surgical excision

**Patient id**	**Age**	**Gender**	**Tumor size (cm)**	**Location**	**Type**	**Local recurrence**	**Metastasis**	**Protocol at relapse**	**DFS (mo)**	**DFS status**	**OS (mo)**	**OS status**
1	19	M	12.00	Pleura	Well-differentiated	Yes	No	S	48	Disease relapse	56	Censored
2	30	F	6.00	Pleura	Well-differentiated	No	No		48	Censored	48	Censored
3	36	F	14.00	Mediastinum	Well-differentiated	Yes	No	S	36	Disease relapse	60	Censored
4	60	M	28.00	Pleura	Well-differentiated	No	No		43	Censored	43	Censored
5	63	M	6.00	Mediastinum	Well-differentiated	No	No		8	Censored	8	Censored
6	72	M	16.00	Mediastinum	Well-differentiated	No	No		48	Censored	48	Censored
7	16	F	7.00	Mediastinum	Myxoid	No	No		36	Censored	36	Censored
8	20	F	4.00	Pleura	Myxoid	Yes	No	S + RT	47	Disease relapse	90	Censored
9	44	M	8.00	Pulmonary	Myxoid	No	No		13	Censored	13	Censored
10	47	M	8.00	Mediastinum	Myxoid	Yes	No	S	12	Disease relapse	27	Dead
11	54	M	16.00	Pleura	Myxoid	No	No		26	Censored	26	Censored
12	61	F	12.00	Pulmonary	Myxoid	No	To liver	CT	3	Disease relapse	14	Dead
13	41	M	16.00	Pleura	Dedifferentiated	Yes	No	S	10	Disease relapse	15	Dead
14	53	M	8.00	Pleura	Dedifferentiated	No	To lung	CT	6	Disease relapse	11	Dead
15	59	F	7.00	Pulmonary	Dedifferentiated	Yes	No	RT	6	Disease relapse	9	Dead
16	64	M	6.00	Mediastinum	Dedifferentiated	No	No		12	Censored	12	Censored
17	49	M	4.50	Pulmonary	Pleomorphic	No	To bone	RT + CT	13	Disease relapse	16	Censored

*M*: male, *F*: female, *S*:surgery, *RT*: radiotherapy, *CT*: chemotherapy.

**Table 2:** Basic characteristics of 6 patients received non-radical surgical excision

**Patient id**	**Age**	**Gender**	**Tumor size (cm)**	**Location**	**Type**	**Metastasis**	**Adjuvant protocol**	**OS (mo)**	**OS status**
1	50	F	8.00	Mediastinum	Well-differentiated	No	No	60	Dead
2	61	M	39.00	Pleura	Well-differentiated	No	No	18	Censored
3	59	F	10.00	Mediastinum	Myxoid	To lung	CT	10	Dead
4	45	F	16.00	Pleura	Myxoid	To lung	CT	7	Dead
5	42	F	8.00	Mediastinum	Pleomorphic	To bone	RT + CT	13	Dead
6	23	F	20.00	Pleura	Pleomorphic	No	RT	14	Dead

### Follow-up

All patients were followed up (follow-up with investigation letter or telephone,) until 29 March 2013. The range of follow-up time was from 7 to 90 months, with median follow-up period of 16 months. At the end of the observation, 10 patients died and 13 patients were still alive.

### Statistical analysis

For survival analysis, OS was measured as time from disease diagnosis to death from tumor, or censoring for patients alive at the time of their final follow-up. DFS was defined as time from complete resection until local recurrence or metastasis. OS and DFS were analyzed by means of Kaplan-Meier method followed with log-rank test. Additionally, for the categorical parameters, Chi-square test was performed. Statistical analyses were carried out with use of SPSS software (version 16.0, Chicago, IL) and R (version 2.15.0, http://www.r-project.org). *P* values less than 0.05 were considered statistically significant.

## Results

### Pathological results

Liposarcoma has been subclassified histologically into well-differentiated, myxoid, dedifferentiated and pleomorphic types by 2012 Soft Tissue Sarcoma of NCCN Clinical Practice Guidelines in Oncology.

Of the 23 patients in the study, 8 cases were well-differentiated liposarcomas. In brief histologic features exhibited large areas of mature adipose tissue, with scarce lipoblasts. Immunohistochemical study showed S-100 was positive in all the 8 cases, while both MDM2 and CDK4 were positive in 6 cases.

The 8 myxoid cases contained a mixture of quite uniform round to oval mesenchymal cells and small, often signet ring lipoblasts in a myxoid stroma with delicate arborizing vasculature and focal mucin pools. Immunohistochemical study showed that in all the 8 cases S-100 was positive, while both Actin and HMB45 were negative.

The 4 dedifferentiated liposarcomas were characterized by areas of well-differentiated liposarcoma showing transition to areas of nonlipogenic spindle cell sarcoma of varying morphology and grade. Immunohistochemical study showed protein S-100 was focally expressed in all the 4 cases, while both MDM2 and CDK4 were positive in 3 cases.

The 3 pleomorphic cases had a predominance of spindle cells, with necrosis and widespread anaplasia. Bizarre lipoblasts were present as were multinucleated floret-like giant cells. Due to lack of obvious value, immunohistochemical was not studied in the 3 cases.

### Imaging findings

Imaging features depend directly on the tumor components and types. Well-differentiated liposarcomas resemble lipomas on both CT and MRI. They contain a large amount of fat, usually more than 75% of their volume. Fibrous septa might be broader and more nodular than those seen in lipomas. Myxoid liposarcomas contain lipoblastic mesenchymal cells and a plexiform capillary network, all set in a myxoid matrix. Because the fat content is often less than 10–25% of the tumor volume, CT might not show the typical features of lipomatous tumors. Pleomorphic and dedifferentiated liposarcomas have the same radiological appearances. Both types usually contain little or no fat. For this reason, it is often impossible to differentiate them from other soft-tissue sarcomas.

MRI represents the gold standard for diagnostic evaluation and preoperative planning due to its superior definition of the tumor invasion of vessels in the mediastinum and the thoracic inlet.

### Treatment methods

Complete surgical excision was attempted in 17 of the total 23 patients. Unfortunately, 9 patients of these 17 patients suffered from relapse. Among these cases, 2 cases of well-differentiated type belonging to mediastinal and pleural groups respectively were local recurrence. 2 cases of myxoid type were local recurrence, each one in mediastinal or pleural group and 1 case of myxoid type with metastases of the liver in pulmonary group. 3 cases of dedifferentiated type were relapsed, including 1 local recurrence in the pleural group and 1 in the pulmonary group, and 1 case in the pleural group with metastases of the lung .1 case of pleomorphic type had metastases of the bone, belonging to pulmonary group. The lymph nodes were negative in all the cases of pulmonary group.

Among above 9 patients, 3 metastatic cases did not respond to chemotherapy or/and radiotherapy. For the remaining 6 local recurrence cases, adjuvant radiotherapy or/and salvage surgery were made depending on the individual situation (Table 1).

In addition, 6 of the total 23 patients received non-radical therapy, including 2 well-differentiated, 2 myxoid and 2 pleomorphic cases (Table 2). The 2 myxoid and 2 pleomorphic patients died approximately one year after surgery. 1 well-differentiated patient died in 60 months, while the other is still alive. It was noted that the myxoid and pleomorphic types basically did not respond to chemotherapy or/and radiotherapy.

### Survival analysis of the patients

The median follow-up time of the 23 patients was 16 months (range: 7 months-90 months). The estimated 2-year DFS and OS rates were 62.70% (95% CI, 42.90-91.80%) and 61.70% (95% CI, 43.90-86.80%) respectively.

**Figure 1:**
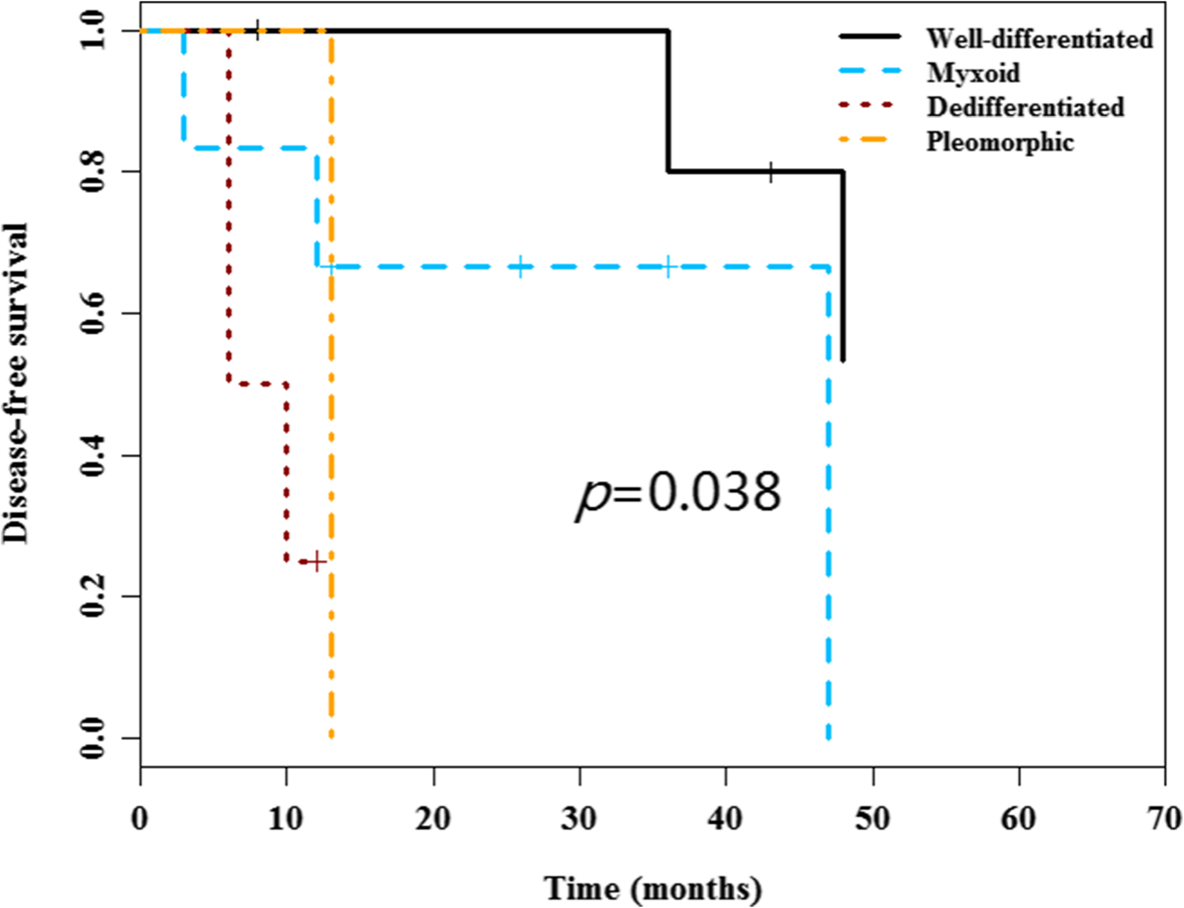
**Disease-free survival curves according to the tumor type.** Poor DFS was observed in the myxoid, dedifferentiated and pleomorphic types as compared to well-differentiated type (*P* = 0.038).

**Figure 2:**
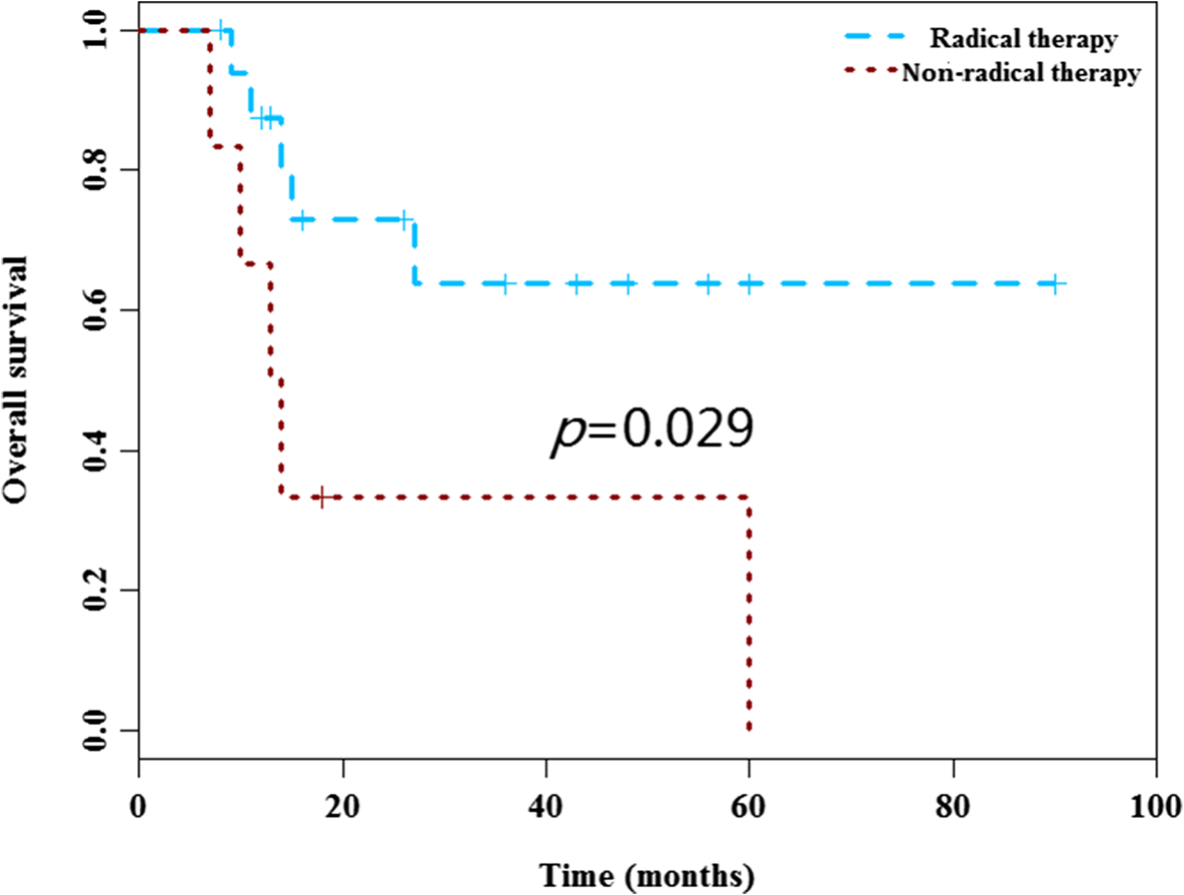
**Overall survival curves according to the tumor type.** Inferior OS were observed in dedifferentiated, myxoid and pleomorphic types, relative to well-differentiated type ( *P* = 0.027).

**Figure 3:**
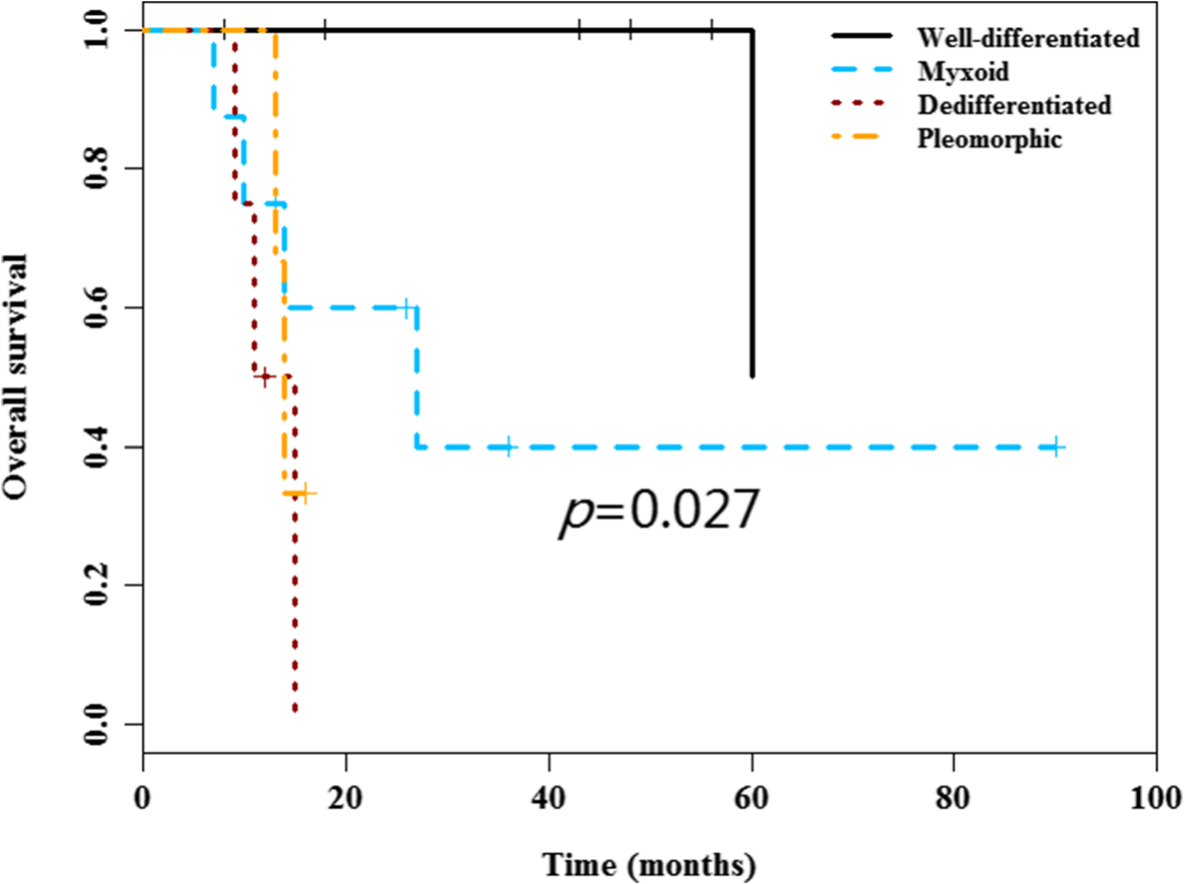
**Overall survival curves according to the surgery.** The variable of surgery was a prognostic factor for OS, as demonstrated by the poor OS in the non-radical surgery group as compared to that of the radical surgery group ( *P* = 0.029).

No significant differences for DFS and OS among distinct tumor locations were observed. Nevertheless, there were significant differences for DFS and OS among distinct liposarcoma types. Poor DFS was observed in the myxoid (median DFS 47 months), dedifferentiated (median DFS 6 months and pleomorphic types (median DFS 13 months) as compared to well-differentiated type (median DFS not reached) (*P* = 0.038) (Figure 1). Inferior OS was observed in dedifferentiated (median OS 11 months), pleomorphic types (median OS 14 months) and myxoid (median OS 27 months), compared to well-differentiated type (median OS 60 months) (*P* = 0.027) (Figure 2). Notably, there were no significant difference for the surgery protocols between distinct liposarcoma types (Chi-square *P* = 0.32 for OS). Additionally, the surgery procedure was a prognostic factor for OS, as demonstrated by the poor OS in the non-radical surgery group (median OS 13 months) as compared to that of the radical surgery group (median OS not reached) (*P* = 0.029) (Figure 3). Notably, there were no significant differences for DFS and OS in other clinical parameters including tumor size, gender and age (Table 3).

**Table 3:** Survival data of the patients classified by distinct factors in the study

**Variable**		**OS**		**DFS**		
	**Case number**	**Median OS (month)**	***P***	**Case number**	**Median DFS (month)**	***P***
Age			0.566			0.516
<= 60 y	18	60		13	47	
> 60 y	5	not reached		4	not reached	
Gender			0.216			0.685
Male	12	not reached		11	48	
Female	11	60		6	36	
Type			0.027			0.038
Well-differentiated	8	60		6	not reached	
Myxoid	8	27		6	47	
Dedifferentiated	4	11		4	6	
Pleomorphic	3	14		1	8	
Location			0.713			0.136
Mediastinum	10	60		6	not reached	
Pleura	9	not reached		7	47	
Pulmonary	4	14		4	6	
Size (ref = less 8 cm)			0.987			0.849
<= 8 cm	12	60		10	47	
> 8 cm	11	not reached		7	36	
Therapy			0.029			
Radical	17	not reached		17	47	
Non-radical	6	13		6*		

*The date of DFS are not included non-radical cases.

## Discussion

Liposarcoma is the most common soft-tissue sarcoma and accounts for approximately 20% of all mesenchymal malignancies [4]. Liposarcomas are usually found in adults and occur rarely in children. About 90–95% of these tumors occur in the trunk, the extremities or retroperitoneum. Primary intrathoracic liposarcomas are unusual, representing 2.7% of all locations [5].

Liposarcomas originate from primitive mesenchymal cells rather than from mature fat cells [6]. Enlarged or irregular adipocyte nuclei, multivacuolated cells, or sclerosing areas are each highly suggestive that the neoplasm in question is indeed a liposarcoma.

Other sarcomas that may be confused with liposarcoma include malignant fibrous histiocytoma, in which bizarre pleomorphic giant cells may be mistaken for pleomorphic liposarcoma. As liposarcoma histology varies, it cannot be often correctly diagnosed by percutaneous biopsy, or even misdiagnosed.

Perhaps because of the expansile rather than infiltrative growth pattern of most of these tumors, patients often presented with few symptoms despite having large or even massive tumors. The mean tumor size in our study is 12.15 ± 8.2 cm. 20 patients presented symptoms usually related to respiratory compromise, including chest pain, cough, and shortness of breath. 3 patients were asymptomatic, including one pleural well-differentiated liposarcoma with the size of 12 cm.

Liposarcomas of the lung and mediastinum have been reported [7], and distinction from pleural liposarcoma with chest wall or mediastinal invasion requires careful radiographic evaluation, surgical evaluation, or both. In some cases it may be impossible to be certain where the tumor is arising.

According to the classification of tumor location, pleural group of our data presented larger tumor size than that of the other two groups. But the tumor size was not the factor of the prognosis (*p* = 0.987). Pleural liposarcoma always reach large proportions because they grow insidiously in areas that are not accessible to physical examination.

Pulmonary lesions are usually metastatic. Metastasis to various structures including lung, pleura, liver and bone are described, specially in the poor differentiated varieties [8]. For only 4 case in all of the pulmonary liposarcoma in our study, one case was still alive after 13 months follow-up without disease, one case had local recurrence, and the other two cases had metastases of liver and bone. But the lymph nodes were negative in all the cases of pulmonary group. This phenomenon implies that liposarcoma is metastasized through blood rather than lymph node.

In previous reports, the clinical behavior of mediastinal liposarcomas has been variable but ultimately fatal in most cases. In Okurnori’s review [9], 10 of 14 patients died after a mean interval of 2.7 years. In Cicciarelli’s series [10], four of seven patients died following resection after a mean of 1.5 years. For 9 of mediastinal liposarcomas in our data, four cases died, in which three cases had non-radical surgery. All the three cases were implied tumor invasion of vessels by MRI before surgery. It is consistent with the result of the previous study that non-radical resection is an unfavorable factor of the overall survival.

Although sometimes the location of the liposarcoma is difficulty to be determined, our data show that there is no difference for DFS (*p* = 0.136) and OS (*p* = 0.713) among the three tumor locations.

Our study demonstrates that the most common histologic types of intrathoracic liposarcoma appear to be well-differentiated (34.78%, 8/23) and myxoid cases (34.78%, 8/23), which is consistent to reports of the literatures. All forms of liposarcoma contain lipoblasts in varying numbers and morphologic forms; however, the more poorly differentiated liposarcomas behave more aggressively and local and distant complications.

Obviously, radical surgery is a favorable prognostic factor as demonstrated by the better OS of patients receiving radical surgery than that of patients receiving non-radical surgery (*P* = 0.029). In the radical group, different types of liposarcomas showed different DFS (*P* = 0.038). In the non-radical group, radical surgery was not performed mainly due to the invasion of tumors into adjacent vital structures, such as heart, great vessels, trachea, bronchi and esophagus. Although the size of the non-radical group tumors are all equal to or larger than the median size of all the tumors, the size is not the factor contributed to incomplete surgical excision in our opinion. Contrast-enhanced chest CT scans and MRI examination are considered important to determine the complete extent of the mass lesion, which has significant influence on surgical planning. In patients where surgical resection is incomplete, a CT scan and MRI frequently defines the size and location of residual tumor.

Well-differentiated liposarcoma is a low or intermediate grade malignancy tumor. In our study, one well-differentiated case died of compressive complication. This patient was performed not radical but non-radical resection due to complete surgical removal was complicated by the lobules of tumor to wrap around great vessels and mainstem bronchi. Notably, the other well-differentiated case receiving non-radical resection without adjuvant therapy, was still alive and without the tumor enlargement after 18 months follow-up. The remaining well-differentiated cases who were performed radical surgery, were all still alive. Our data demonstrated the well-differentiated type is a good prognostic factor.

In our study, among 8 myxoid cases, 4 cases died. One cases died of metastases of liver, two cases died of metastases of lung, the other one died of compressive complication.

Our data show myxoid liposarcoma was prone to metastase and basically had no chemotherapy or/and radiotherapy response, except local recurrence of radical surgery case had salvage surgery and radiotherapy, he was still alive.

Radiotherapy had also been of some value as an adjuvant treatment for the myxoid type [11-13]. Since the limited data, we didn’t draw the conclusion.

The pleomorphic and de-differentiated types are regarded as highly malignant tumors with a tendency towards local recurrence and metastasis.

Two patients had non-radical resection and one patient had distant bone metastases in all three pleomorphic liposarcoma. Although the bone metastases case was still alive, the patient was followed up only 3 months, and the bone lesion was not improved by therapy. Three patients relapsed after radical surgery in four dedifferentiated liposarcoma and they all die. Totally seven patients of the two types cases, five patients had died about 1 year after operation, while the other two patients were still alive with and without disease respectively. Our data provides additional evidence that the pleomorphic and de-differentiated types are unfavorable prognostic factors. Besides, our result suggest these two types of liposarcomas also had no chemotherapy or/and radiotherapy response.

The result of our study showed the histologic types of the liposarcoma is an important prognosis factor of OS (*P* = 0.027). Notably, there were no significant difference for the surgery procedure between distinct liposarcoma types (Chi-square *P* = 0.32 for OS). Therefore, the histologic types is a potential independent prognostic factor.

The use of adjuvant treatment for intrathoracic liposarcoma is controversial. Doxorubicin and ifosfamide are the most frequently used chemotherapeutic agents in patients with intrathoracic liposarcoma [14].

Hirai et al. [15] reported in general, radiotherapy and chemotherapy are believed to be ineffective therapeutic modalities for survival. At present, therefore, the best treatment for mediastinal liposarcoma should be complete surgical resection.

In our medical center, Doxorubicin and ifosfamide are also the most commonly used chemotherapeutic agents. Evidence from our medical center cases reviewed here indicates that adjuvant radiotherapy or/and chemotherapy may not benefit patients with primary intrathoracic liposarcoma. But the limitation presented in this study must be considered. The population in this study was small because the disease is rare. Future studies with larger patient population is needed, and whether radiology and chemotherapy seem to be effective treatment as induction therapy can be also investigated.

## Conclusions

There were no significant differences for survival among distinct liposarcoma locations. Nevertheless, liposarcoma types were associated with the survival. The well-differentiated liposarcoma patients who received radical surgery had the best prognosis. The histological type of the liposarcoma and the radical surgery are the factors that influence tumor behaviour and prognosis. In general, radiotherapy and chemotherapy are believed to be ineffective therapeutic modalities for survival. So we should try our best to completely resect the primary intrathoracic liposarcoma.

### Consent

Written informed consent was obtained from the patient for the publication of this report and any accompanying images.

## Abbreviations

CT: Computed tomography; MRI: Magnetic resonance imaging cm: Centimeter.

## Competing interests

The authors declare that they have no competing interests.

## Authors’ contributions

MC and JY performed surgery. LZ carried out the patient diagnosis. MC and JY were major contributors in writing the manuscript. HZ Provide a lot of useful suggestions about this manuscript. CZ did a lot of work to collect data. All authors read and approved the final manuscript.

## References

[B1] Carroll F Kramer MD Acinapura AJ Tietjen PA Wagner I Oiseth S Smith F Pleural liposarcoma presenting with respiratory distress and suspected diaphragmatic hernia Ann Thorac Surg 1992 9 1212 1213 10.1016/0003-4975(92)90102-A 1449314

[B2] Wong WW Pluth JR Grado GL Schild SE Sanderson DR Liposarcoma of the pleura Mayo Clin Proc 1994 9 882 888 10.1016/S0025-6196(12)61792-3 8065192

[B3] Sekine Y Hamaguchi K Miyahara Y Baba M Yasufuku K Fujisawa T Yamaguchi Y Thymus-related liposarcoma:report of a case and review of the literature Surg Today 1996 9 203 207 10.1007/BF00311509 8845616

[B4] Enzinger F Weiss SW Liposarcomas Soft Tissue Tumors 1995 3 St. Louis: CV Mosby 431 466

[B5] Shmookler BM Enzinger FM Liposarcoma occuring in children.an analysis of 17 cases and review of the literature Cancer 1983 9 567 574 10.1002/1097-0142(19830801)52:3<567::AID-CNCR2820520332>3.0.CO;2-4 6861094

[B6] Sung MS Kang HS Suh JS Lee JH Park JM Kim JY Lee HG Myxoid liposarcoma: appearance at MR imaging with histologic correlation Radiographics 2000 9 1007 1019 10.1148/radiographics.20.4.g00jl021007 10903690

[B7] Gordon MS Hajdu SI Bains MS,15, Burt ME. Soft tissue sarcomas of the chest wall: results of surgical resection J Thorac Cardiovasc Surg 1991 9 843 854 2023441

[B8] Sawamura K Hashimoto T Nanjo S Nakamura K Iioka S Mori T Furuse K Shakudo Y Primary liposarcoma of the lung; report of a case J Surg Oncol 1982 9 243 246 10.1002/jso.2930190414 7078177

[B9] Okurnori M Mabuchi M Nakagawa M Malignant thymoma associated with liposarcoma of the mediastinum. A case report Jpn J Surg 1983 9 512 518 10.1007/BF02469495 6672382

[B10] Cicciarelli FE Soule EH McGoon DC Lipoma and liposarcoma of the mediastinum: a report of 14tumors including one lipoma of the thymus J Thorac Cardiovasc Surg 1964 9 411 14180746

[B11] Ibe T Otani Y Shimizu K Nakano T Sano T Morishita Y Pulmonary pleomorphic liposarcoma Jpn J Thorac Cardiovasc Surg 2005 9 443 447 10.1007/s11748-005-0082-y 16164258

[B12] Krygier G Amado A Salisbury S Fernandez I Maedo N Vazquez T Primary lung liposarcoma Lung Cancer 1997 9 271 275 10.1016/S0169-5002(97)00030-5 9237162

[B13] Loddenkemper C Perez-Canto A Leschber G Stein H Primary dedifferentiated liposarcoma of the lung Histopathol 2005 9 710 712 10.1111/j.1365-2559.2005.02041.x 15910606

[B14] Mikkilineni RS Bhat S Cheng AW Prevosti LG Liposarcoma of the posterior mediastinum in a child Chest 1994 9 1288 1289 10.1378/chest.106.4.1288 7924520

[B15] Hirai S Hamanaka Y Mitsui N Uegami S Matsuura Y Surgical resection of primary liposarcoma of the anterior mediastinum Ann Thorac Cardiovasc Surg 2008 9 1 38 41 18292740

